# The potential of developing a protective peptide‐based vaccines against SARS‐CoV‐2

**DOI:** 10.1002/ddr.21969

**Published:** 2022-06-25

**Authors:** Ahmed O. Shalash, Istvan Toth, Mariusz Skwarczynski

**Affiliations:** ^1^ School of Chemistry and Molecular Biosciences The University of Queensland St. Lucia Queensland Australia; ^2^ School of Pharmacy The University of Queensland Woolloongabba Queensland Australia

**Keywords:** efficacy, genetic vaccines, peptide vaccines, safety, SARS‐CoV‐2

## Abstract

COVID‐19 pandemic has been the deadliest infectious disease outbreak since Spanish flu. The emerging variant lineages, decay of neutralizing antibodies, and occur of reinfections require the development of highly protective and safe vaccines. As currently approved COVID‐19 vaccines that utilize virus‐related genetic material are less than ideal, other vaccine types have been also widely investigated. Among them, peptide‐based vaccines hold great promise in countering COVID‐19 as they may overcome most of the shortcomings of RNA/DNA and protein vaccines. Two basic types of potential peptide vaccines can be developed. The first type are those which rely on cytotoxic T‐cell (CTL) responses to kill infected host cells and stop the replication via employing CTL‐epitopes as vaccine antigens. The second type of peptide vaccines are those that rely on B‐cell peptide epitopes to trigger humoral response via generating SARS‐CoV‐2‐specific antibodies to neutralize and/or opsonize the virus. We propose that combining both cellular and humoral immune responses would be highly protective. Here we discuss opportunities and challenges in the development of an effective and safe peptide‐based vaccine against COVID‐19.

1

As of October 2021, severe acute respiratory syndrome coronavirus 2 (SARS‐2), causative agent of COVID‐19, has resulted in over 240 million infections worldwide and 5 million deaths (Dong et al., 2020). In addition to the overall infection rate, the rapid decay of neutralizing antibodies (Abs) in convalescent patients' (CP) serum (Bölke et al., 2020), reinfection occurrence (Iwasaki, 2021), increased virulence of emerging lineages, and future virus spillover from animal reservoirs necessitate urgent development of an effective and safe vaccine (Shalash et al., 2021b). The virus infects lower airway tissues, where pneumocytes‐II bearing angiotensin converting enzyme‐2 receptor (ACE2) are located. The virus surface protein, spike protein (SARS‐2‐S), binds to host ACE2 receptors via the receptor binding domain (RBD), which allows virus entry into host cells to replicate.

Design of peptide vaccines against COVID‐19 has been greatly inspired by vaccine development against SARS‐CoV (SARS‐1), the causative agent of SARS pandemic in 2003. For example, Wang et al. studied SARS‐1‐S RBD‐derived peptide vaccines which reduced viral lung titers by 20 folds and decreased pneumonia (Wang et al., 2016). In addition, mice immunized with SARS‐1‐S‐derived cytotoxic T‐cell (CTL) epitopes were protected from lethal SARS‐1 infection challenge (90–100%) and had reduced viral titers (~10^3^ folds) (Channappanavar et al., 2014). Thus, T‐helper and CTL epitope antigens should be explored further in coronavirus vaccine development.

In the case of SARS‐2, the vaccine development has relied mainly on genetic vaccines. RNA‐based vaccines typically encode SARS‐2‐S. In vivo expression in host cells of SARS‐2‐S ensures its proper folding/conformation and glycosylation. RNA vaccines also trigger cytoplasmic pathogenic recognition receptors that help trigger Th1 responses, such as retinoic acid‐inducible gene I and toll‐like receptors (Pulendran et al., 2021). In contrast, DNA vaccines trigger considerable side effects and change transfected cells' genetic material content (Ramasamy et al., 2021). RNA vaccines do not carry this risk, and the possibility of reverse transcription of vaccine RNA has been disproven (Parry et al., 2021). The selection of specific immunogenic and neutralizing subdomains within SARS‐2‐S sequence could minimalize side‐effects through omission of dangerous sequences, e.g., BNT162b1 RNA vaccine only encodes the RBD sequence (Sahin et al., 2020). However, genetic vaccines are expensive and pose critical hurdles in terms of stability, cryostorage and transport, and side effects from live, or nonlive cationic, vectors (Ramasamy et al., 2021; Sahin et al., 2020; Shalash et al., 2021b). Furthermore, it has been demonstrated that most broadly used RNA‐vaccine (BNT162b2) protection is short‐lived; initial efficacy against SARS‐2 infection (88%) has been reduced to just 47%, 5 months post‐immunization (Long et al., 2020; Tartof et al., 2021).

As SARS‐2 genetic vaccines are less than ideal, other vaccines types have been also widely investigated, including SARS‐2‐S protein vaccines. When the immunogenicity of SARS‐2‐S adjuvanted with alum/CPG was investigated in a clinical study against SARS‐2, severe systemic and local side effects were reported (Richmond et al., 2021). In contrast, Novavax®, a matrix‐M‐adjuvanted recombinant full‐length SARS‐2‐S vaccine, demonstrated good efficacy (89%) and better tolerability in phase 3 clinical trials (Heath et al., 2021). However, full‐length SARS‐2‐S might still not be the ultimate antigen due to difficulties in stabilizing its desired prefusion conformation, and the presence of immunopathological sequences (Mortaz et al., 2020; Shalash et al., 2021b). In addition, off‐target dose loss of SARS‐2‐S due to ACE2 binding in non‐immune cells has also been overlooked (Figure 1) (Shalash et al., 2021b).

**Figure 1:**
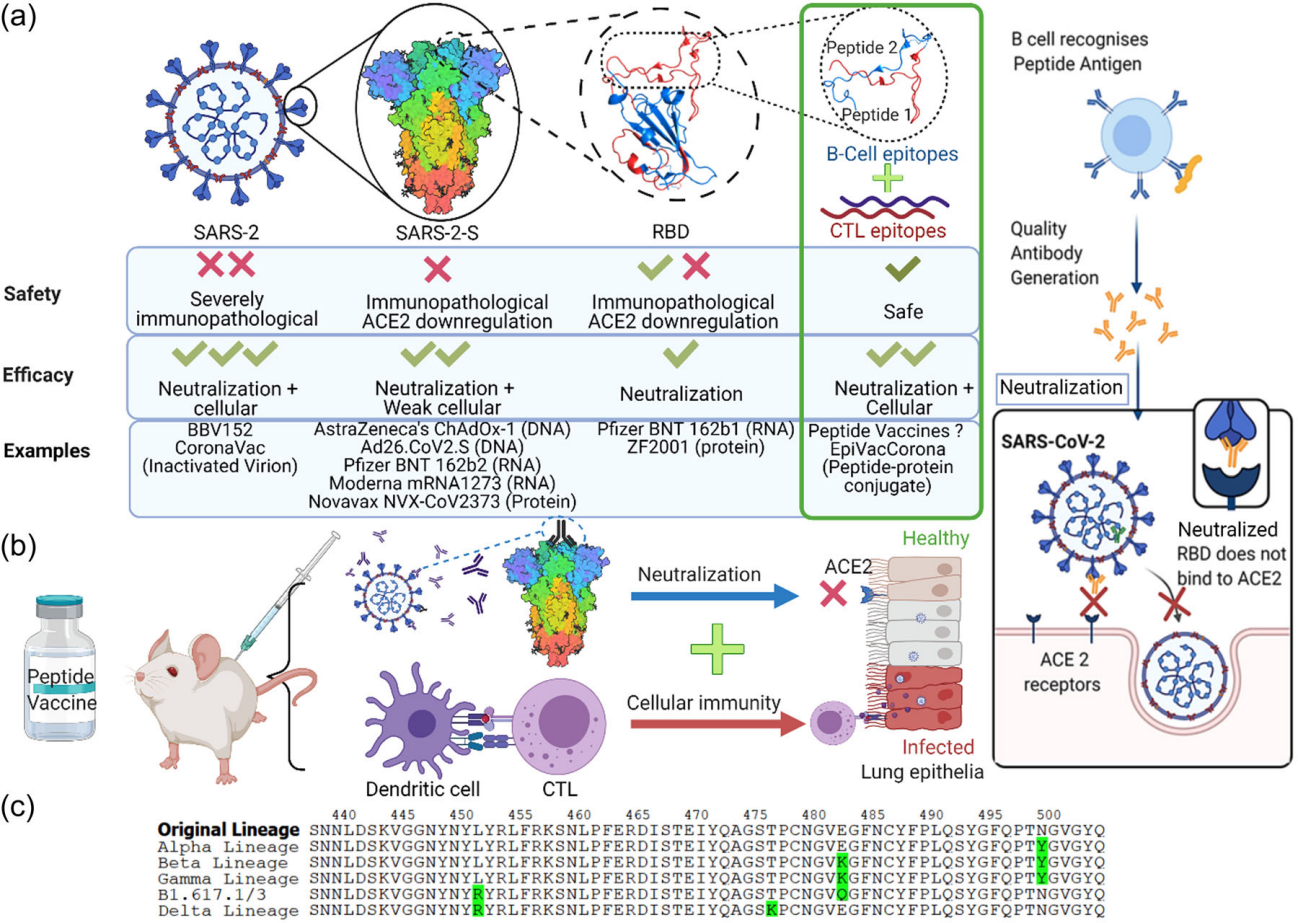
Schematic representation of the subunit peptide vaccine strategy of clinically investigated and/or approved vaccines (a); illustration of protective mechanisms (b); and RBM sequences of emergent variant lineages with mutant residues highlighted in green (c). RBM, receptor binding motif

Currently approved subunit vaccines rely only on SARS‐2‐S, or its fragments, and are expected to trigger mostly humoral immunity, thus neutralizing and opsonizing antibody‐based protection, rather than CTL‐based immune responses. However, many of the CTL epitopes recognized by human MHC‐I alleles were identified in proteins other than SARS‐2‐S (Shalash et al., 2021b; Shomuradova et al., 2020). Unfortunately, these highly protective, conserved, CTL epitopes from other SARS‐2 proteins have not been yet incorporated in currently available SARS‐2 vaccines.

Peptide‐based vaccine against SARS‐2 can be designed by combining B‐ and T‐cell epitopes from different viral proteins, including non‐structural proteins. For example, B‐cell epitopes can be derived from the receptor binding motif (RBM), while T‐cell epitopes can be chosen from variety of already identified SARS‐2 T‐cell epitopes (Shomuradova et al., 2020). The relative efficiencies of T‐cell and B‐cell epitopes have recently been reviewed (Shalash et al., 2021b). Unfortunately, determination of the correlation between T‐cell immunity and protection against SARS‐2 is progressing slowly compared to the correlation of neutralizing B‐cell responses with protection. Hopefully, humanized mice infection challenge can reveal the protective efficacy of these epitopes.

One crucial source of B‐cell epitopes is the RBM—the fragment of the RBD that is in close contact with ACE2. Several of the highly potent neutralizing Abs, IC_50_ = 5–10 ng/ml (Liu et al., 2020), obtained from CP sera were directed against epitopes within the RBM sequence, especially residues S^445–500^ (Shalash et al., 2021b). In contrast, most of the neutralizing Abs in CP sera that were directed against N‐terminus terminal domain (NTD) epitopes were of lower potency (Liu et al., 2020). Further, several neutralizing Abs that were directed against RBM remained potently neutralizing against emerging SARS‐2 variant lineages (Stamatatos et al., 2021), as only four RBM residues, R^452^L, K^478^T, K/Q^484^E, and N^501^Y, were altered in emergent variant lineages (Figure 1c). The RBM‐derived epitopes were also found to be neutralizing (e.g., SARS‐2‐S^451–470^ and S^491–510^) in mice when conjugated to diphtheria toxoid and adjuvanted with alum or emulsion‐based adjuvants (Pandey et al., 2021; Shalash et al., 2022). When sera of mice immunized with two different epitopes were combined, synergistically inhibited RBD/ACE2 binding when examined using competitive ELISA (Pandey et al., 2021; Shalash et al., 2021a). Furthermore, 14–24‐mer peptides were used as vaccine antigens to identify neutralizing epitopes in BALB/c mice. NTD‐epitopes (SARS‐2‐S^63–85^ and S^92–106^), and RBM‐derived epitopes (SARS‐2‐S^439–454^, S^455–469^, and S^475–499^) were found to be strongly neutralizing against the original and D614G SARS‐2 strain (Lu et al., 2021). Short RBD‐derived epitopes were considered to be of insufficient length to produce potently neutralizing nAbs, which have only been observed with protein/RNA subunit vaccines so far. Therefore, longer peptides could be employed to provide the highly discontinuous epitopes needed to trigger the production of potently neutralizing Abs (Shalash et al., 2022; Shalash et al., 2021b). Furthermore, recently complete Freund (CFA)‐adjuvanted, long RBM‐derived, peptide epitope (S^444–483^) demonstrated potent neutralization (serum nAb titers ≈300), against S‐protein pseudotyped‐virions, which was equivalent to CFA‐adjuvanted RBD protein in BALB/c mice (Shalash et al., 2022). EpiVacCorona (Vektor State Research Centre, Russia) is a peptide vaccine composed of three short peptides derived from SARS‐2‐S (S^454–478^, S^1181–1202^, and S^1191–1211^) conjugated to SARS‐2 nucleocapsid protein. The vaccine induced the production of nAb titers of about 40, following immunization in ferrets. Despite modest neutralizing Ab titers, the efficacy was attributed to the synergistic protection offered by T‐cell immunity and strong opsonic Abs (Ryzhikov et al., 2021a, 2021b). EpiVacCorona induced seroconversion in all volunteers and moderate nAb titers approximately 20 in phase 1/2 clinical trials (Ryzhikov et al., 2021b). There are currently no approved peptide vaccines against SARS‐CoV‐2, however, several peptide vaccine candidates are currently undergoing clinical trials (Table 1).

**Table 1:** Peptide vaccines in clinical trials on healthy adult volunteers

Vaccine	Status	Outcomes	Trial number
CoVePiT 3 (OSE Immunotherapeutics, Belgium) Antigen: Conserved CTL‐epitopes from 11 SARS‐CoV‐2 proteins	Phase 1 Recruiting	N/A	NCT04885361
EpiVacCorona (Vector Institute, Russia) Antigen: RBD‐derived neutralizing peptide epitopes conjugated to N‐protein	Phase 1/2 Completed	About 79% of volunteers seroconverted. However, neutralization efficacy assay results were not reported.	NCT04527575
	Phase 3 Completed	N/A	NCT04780035
naNO‐COVID (Emergex Vaccines, Switzerland) Antigen: SARS‐CoV‐2‐derived T‐cell epitopes loaded onto gold nanoparticles	Phase 1 Recruiting	N/A	NCT05113862
pVAC/CoVac‐1 (University Hospital Tuebingen, Germany) Antigen: SARS‐CoV‐2‐derived T‐cell epitopes Adjuvant: TLR1/2 ligand XS15 and Montanide ISA 51	Phase 1 Completed	Highly tolerable and safe. IFN‐γ ELISPOT assay showed stronger activation of CD4+ and CD8+ T‐cell responses in all participants, compared to those reported by mRNA vaccine (Heitmann et al., 2022).	NCT04546841

Abbreviations: mRNA, messenger RNA; RBD, receptor binding domain; SARS‐CoV‐2, severe acute respiratory syndrome coronavirus 2.

Vaccine antigen selection should not only focus on original SARS‐2 lineage sequences, but also consider new mutant variant sequences; especially, as several mutations have increased the virulence and also have compromised the efficacy of approved vaccines. Several in vitro methods have been employed to evaluate SARS‐2‐S neutralization efficacy (Shalash et al., 2021a, 2021b; Shalash et al., 2022), and these methods can be adapted to evaluate vaccine efficacy against emerging variants. Further, since the immune correlates of protection of several approved vaccines were evaluated in animal infection challenge models (e.g., ferrets and non‐human primates) and in humans, validation of a translation model is possible to infer higher confidence in the relevance of animal infection challenge outcomes to efficacy in humans (Shalash et al., 2021b). An example of such valuable relationships and their applications include the reported relationship between nAb titers and anti‐RBD immunoglobulin G (IgG) titers in the sera that was generated by BNT‐621 messenger RNA (mRNA) vaccines in adult volunteers (Mulligan et al., 2020; Shalash et al., 2021b), ${\mathrm{Log}}_{10}\mathrm{nAb}=1.53+0.94\times {\mathrm{Log}}_{10}{\mathrm{IgG}}_{\mathrm{anti}-\mathrm{RBD}}$. This relationship shows that neutralization essentially begins after exceeding a minimum threshold of serum log_10_ anti‐RBD IgG titers of about 1.53, and then neutralization increases with anti‐RBD titers with a slope of about 0.94 beyond this threshold value. Therefore, to achieve similar or higher neutralization values to those of convalescent COVID‐19 patients, who have nAb titers of about 100, the minimum target level of immunogenicity for the mRNA vaccine would be above log_10_ anti‐RBD IgG titers of 2.6 in healthy adult serum. Similar approaches that rely on reported efficacy outcomes can be employed to obtain similar relationships. Additional valuable relationships could be established via correlation of efficacy results among different animal infection‐challenge models and humans, thus establishing a translational model that could potentially predict cross‐species efficacy for future vaccine development. Furthermore, Immune correlates of “cellular response‐based” protection have been overlooked, thus, similar inter‐, and intra‐species, efficacy evaluations of cellular responses to immunization should established in the future.

Peptide‐based vaccines hold great promise in countering SARS‐2 infections. They inherently overcome most of the shortcomings of RNA and protein vaccines. By selecting minimally immunogenic and neutralizing component(s), we (a) avoid immunopathological sequences; (b) focus the immune response on neutralizing humoral responses; and (c) prevent off‐target loss of antigen dose and promotion of lung injury due to downregulation of ACE2 (Shalash et al., 2021b). Moreover, the RBD secondary structure is predominantly random coil, which can be easily adopted by peptides. Other advantages of the peptide‐based approach include ease of chemical synthesis at massive scale, avoidance of biological contaminants, and stability as dry powder under normal storage conditions (M. Skwarczynski & Toth, 2016). The only drawbacks to peptide‐based vaccines are difficulty in generating highly discontinuous neutralizing Abs that are directed against two‐neighboring RBDs, such as those generated against native trimeric SARS‐2‐S (Shalash et al., 2021b). In addtion peptide vaccines have lower immunogenicity compared to protein vaccines (M. Skwarczynski & Toth, 2016). However, low immunogenicity, and even restoration of native conformation, have been overcome in peptide vaccine formulations through combination with approved commercial adjuvants or conjugation to adjuvanting moieties (M. Skwarczynski & Toth, 2016). Conjugation of hydrophobic adjuvanting moieties, such as peptides (Mariusz Skwarczynski et al., 2020) and polymers (Nevagi et al., 2019) to peptide antigens have been proven to greatly improve vaccine efficacy, even when delivered via oral and intranasal routes (Faruck et al., 2020; M. Skwarczynski & Toth, 2016), thus mimicking natural infection and easing the logistics of vaccine distribution and immunization. Although little investigation has gone into developing peptide vaccines against SARS‐2 to date, they may yet show great potential to provide high prophylactic or even therapeutic efficacy.

## CONFLICTS OF INTEREST

The authors declare no conflicts of interest.

## Data Availability

Data sharing is not applicable to this article as no new data were created or analyzed in this study.
